# Microhardness of glass carbomer and high-viscous glass Ionomer cement in different thickness and thermo-light curing durations after thermocycling aging

**DOI:** 10.1186/s12903-019-0973-4

**Published:** 2019-12-04

**Authors:** Mehmet Buldur, Emine Sirin Karaarslan

**Affiliations:** 1 Department of Restorative Dentistry, Faculty of Dentistry, Çanakkale Onsekiz Mart University, Çanakkale, Turkey; 2 Department of Restorative Dentistry, Faculty of Dentistry, Tokat Gaziosmanpaşa University, 60150 Tokat, Turkey

**Keywords:** Glass carbomer cement, Equia forte, Microhardness, Thermocycling, SEM

## Abstract

**Background:**

The objective of our study was to compare the upper and lower surface microhardness and surface changes of Glass Carbomer Cement (GCP) and EQUIA Forte (EF) in different thickness after thermo-light curing durations and aging.

**Methods:**

A total of 504 samples (5 mm-diameter) were prepared by using GCP-252 (GCP Dental, and Vianen, Netherlands) and EF-252 (EQUIA Forte, GC, Tokyo, Japan). Three different thickness samples (2, 4, and 6 mm) were prepared with 84 samples in each subgroup. The samples were prepared by three curing procedures (Non-exposed, 60s, 90s). Their varnishes were applied to the upper surfaces of half of each subgroup (*n* = 7). The upper microhardness measurements were evaluated before and after aging. To compare the effect of different thicknesses, the bottom surfaces of the samples were evaluated before aging in terms of microhardness measurements. Also, the upper surfaces were analyzed in the SEM before and after aging.

**Results:**

The upper surface values of all the samples were higher than the bottom values (*p* < 0.05). There were no significant differences between the varnished and non-varnished samples in both materials (*p* > 0.05). Although this increase was not significant in some groups, temperature variations increased the surface microhardness values of both materials except for the non-exposed-varnished EF samples. The highest microhardnesses values were recorded in the non-exposed-varnished EF (125.6 ± 6.79) and unvarnished GCP (88.1 ± 7.59) samples which were thermo-light cured for 90 s before aging. The bottom hardness values were affected by thickness variations in both GCP and EF materials (*p* < 0.05). The sample deformations and microcracks after aging were greater than before in all the materials. Thermo-light curing in 90 s to the samples reduced the cracks in both the materials before and after aging.

**Conclusions:**

Thermal aging adversely affected the microhardness of the materials, which is important for clinical success. The thermo-light curing process improved the microhardness of the GCP group without varnish application. Varnish application increased the microhardness of the EF group without applying thermo-light curing. The microhardness of the bottom surfaces decreased with increasing thickness. The thermo-light curing did not increase the bottom surface microhardness of all the samples.

## Background

Glass ionomer cement (GIC) has anticariogenic properties such as damp enamel, dentine adhesion, and long-term fluoride release [1, 2]. Other clinical advantages, including biocompatibility and a low value of thermal expansion, increase the frequency of GIC preference in daily dental use [3–5]. However, they have weak mechanical properties such as poor surface properties, high roughness, and surface wear [6, 7]. The major disadvantages of conventional GIC include their low physical properties such as susceptibility to moisture, opacity [8], and slow curing [9]. The use of conventional GIC as a permanent filler material is not recommended in areas where posterior teeth may be exposed to high stress because of low mechanical properties [10, 11].

GICs are very susceptible to dehydration in the first 10 min following curing, while they are also highly susceptible to dehydration during long-term curing [12]. To maintain water balance in cement, the surface of the newly completed restoration must be protected against water loss as well as water gain [13]. Surface coatings—including softeners such as cocoa butter and petroleum jelly, waterproof varnishes, and methyl methacrylate—amides, and light-curing resins can help overcome this major clinical problem [14, 15]. Varnish application is a common protective approach for GIC. In particular, resin-based coatings are effective in reducing initial water contamination, which plays an important role in the maturation and improvement of the mechanical strength of the material [16].

To improve the mechanical properties of GIC, it is recommended to apply heat during the setting reaction [17, 18]. In particular, heat transfer from light sources to the surfaces of GIC reduces the viscosity of the material by causing increased ion mobility in the initial hardening reaction of the material [19]. The heat applied to GIC during curing allows the cement to rapidly pass through the early stages of curing, where they are most sensitive to moisture. In other words, heat reinforcement reduces premature failure of restoration resulting from overloading of the material before reaching the full strength [20]. When the temperature of the heat applied to the material is high, the powder/liquid ratio may increase because of the vaporization of the liquid, which in turn increases the strength of the cement [9].

The surface hardness value of tooth-colored materials is evaluated to estimate their durability. These mechanical properties also show the relationship among the content of the filler material, the size of the filler material, and the silane coupling agent [21, 22]. The Vickers hardness test is used to measure the surface hardness using a pyramidal recess with a specific load and application time; it is used for brittle materials [23].

GCP (GCP Dental, Ridderkerk, Netherlands) is a new filler material containing nanofluoride hydroxyapatite particles [24]. Due to the nanoparticulate structure of the material, the contact between liquid and powder particles is increased, thereby increasing the hardness and mechanical properties of the filler. However, a strong light source must be used to cure the GCP filler material [20]. Equia Forte (EF; GC, Tokyo, Japan), a highly viscous hybrid derivative of GIC, is a restorative filler material with higher-molecular-weight acrylic acid molecules containing smaller and more reactive silicate particles, which therefore increses the number of cross-links in the matrix structure. It is used in one stage and suggested by the manufacturer to be used as a permanent restorative material in interfacial cavities that are not exposed to high occlusal stresses in the posterior region. It is also recommended to be used as a permanent restorative material on the root surface and in class V cavities [25].

In this study, the aim was to determine the upper surface of materials with different curing procedures and to find out how the bottom surface hardness values were affected against the thermal changes with different thickness.

According to the null hypothesis of the study:

1. There would be no difference between the two different permanent restorative materials to be used in the study in terms of surface hardness values before and after thermal aging.
2. Different thermo-light curing procedures would not change the surface hardness values of the GCP and EF samples.
3. Different thicknesses would not affect the bottom surface hardness values of the GCP and EF samples.

## Methods

The restorative materials and varnish systems used in the study are provided in Table 1. For the preparation of the disk-shaped samples, metal molds having a diameter of 5 mm and depths of 2, 4, and 6 mm were used. There were 504 samples in total and 36 subgroups, including two different types of cement with varnished and unvarnished, three different depths, and three different thermo-light curing periods (none, 60 s, and 90 s) (*n* = 7) (Diagram 1).

**Table 1:** Materials and properties used in this study

Material	Manufacturer	Chemical Component	LOT Number
GCP (Glass Carbomer Cement)	GCP Dental, Vianen, Netherlands	Floralumina silicate glass, apatite, polyacids.	7601837
Equia Forte	GC, Tokyo, Japan	Floralumina silicate glass, carboxylic acid, polyacrylic acid, water.	160119A
GCP Gloss	GCP Dental, Vianen, Netherlands	Modified polysiloxanes	1407106
Equia Forte Coat	GC Europe, Leuven, Belgium	Methyl methacrylate, colloidal silica, camphorquinone, urethane methacrylate, phosphoric ester monomer.	1512051

### Sample preparation

The disk-shaped samples used in the study were prepared by the same operator at room temperature of 23 °C ± 1 °C and relative humidity 50% ± 5% in experimental conditions. A transparent matrix tape (ESR-P universal strip) was placed on a sterile glass, and then the prepared metal mold was placed over it. The GIC filler materials to be tested were injected into the molds after mixing in a mixing device (Functional Capsule Mixer, Monitex Industrial Co. Ltd., New Taipei City, Taiwan) following the manufacturer’s instructions (GCP: 15 s, EF: 10s). Using CarboLED (1400 mW/cm^2^; GCP Dental, Ridderkerk, Netherlands) perpendicular to the material, the samples were prepared in 3 different subgroups., including two groups in which light cure for 60 s, 90 s was applied and light cure was not applied. The tip of the thermo-light curing device was measured at the sensor in the charging chamber, and the light intensity was determined to be the required level. The samples were removed from the metal molds after 5 min, and their thickness was measured with calipers. GCP Gloss for GCP (Glass Carbomer Cement) and Equia Forte Coat for Equia Forte were used in the varnish samples (*n* = 7). Also, after applying the Equia Forte Coat in accordance with the manufacturer’s instructions, additional light for 20 s was applied with CarboLED to the samples of the EF group. The samples were stored in distilled water at 37 °C for one month.

Each sample was embedded in acrylic blocks and cured overnight, with the surfaces to be measured exposed. Surface-finishing operations were carried out on the surfaces of the samples for polishing and leveling for 20 s with silicon carbide disks numbered 400, 800, and 1200, respectively (Metkon Gripo 2 V Grinder/Polisher, Metkon Instruments Inc., Bursa, Turkey).

### Vickers hardness number measurements

Vickers hardness number (VHN) measurements were made on the surfaces of the prepared samples by applying 980 mN pressure for 10 s using a high-quality microhardness tester (Matsuzawa MHT2, Matsuzawa SEIKI Co. Ltd. Tokyo, Japan). Microhardness was measured twice from the center of each sample. Microhardness was calculated using the following formula: HV = 1.8544xF/d2, where d is the diagonal of the imprint, and F = m × g (g = 9.81 N/kg) [26].

### Thermal aging

The samples were subjected to thermal aging (Thermocycler THE-1100, SD Mechatronik, Feldkirchen-Westerham, Germany). The samples were thermocycled 10,000 times between water baths at 5 °C and 55 °C, with a dwell time of 30 s and transfer time of 5 s in each bath. After thermocycling, the samples were subjected to VHN measurements again. The data were calculated as hardness numbers and accordingly plotted as hardness versus depth profiles.

### Scanning Electron microscope analysis

The surfaces of the samples were sputter-coated with gold to a layer of thickness approximately 60 Å in a vacuum evaporator coater (SC7620 Mini Sputter Coater, Quorum Technologies Ltd., West Sussex, United Kingdom). The upper surface topography of the 2-mm-thick samples was examined under a scanning electron microscope (SEM; JSM-6390LV, JEOL Ltd., Tokyo, Japan) at 500× and 1000× magnifications and 20 kV of accelerating voltage.

### Statistical analyses

Statistical analyses were performed using SPSS v.19 (IBM SPSS Statistics for Windows, IBM Corp, Armonk, NY) program. When the effects of factors on pre- and post-aging measurements were explored, variance analysis was used in repeated measures. The Bonferroni correction was used for multiple comparisons. *P*-value of < 0.05 was considered to be statistically significant.

## Results

According to the statistical analyses, inter-and intragroup comparisons are provided in Table 2. Thermal aging resulted in statistically reduced hardness values in both sample groups (*p* < 0.05). The surface hardness values were significantly affected by sample thicknesses. In addition, material differences significantly affected surface microhardness values *(p* < 0.05). However, varnish application and temperature variations did not significantly affect the bottom and upper surface hardness values of both materials (*p* > 0.05), (Table 2).

**Table 2:** Analysis of variance

	Sum of Squares	SD	Averages of Squares	F	P
Material	521447.776	1	521447.776	3684.392	<0.001
Varnish	3.265	1	3.265	0.023	0.879
Thickness	4329.522	2	2164.761	15.296	<0.001
Heat	601.633	2	300.816	2.125	0.121
Surface	45731.135	1	45731.135	323.122	<0.001
Material * Varnish	721.073	1	721.073	5.095	0.024
Material * Thickness	34.401	2	17.2	0.122	0.886
Material * Heat	2577.698	2	1288.849	9.107	<0.001
Material * Surface	4320.844	1	4320.844	30.53	<0.001
Varnish * Thickness	316.89	2	158.445	1.12	0.327
Varnish * Heat	3070.458	2	1535.229	10.847	<0.001
Varnish * Surface	1546.56	1	1546.56	10.928	0.001
Aging	24352.822	1	24352.822	16046.046	<0.001
Aging * Material	1791.061	1	1791.061	1180.128	<0.001
Aging * Varnish	21.624	1	21.624	14.248	<0.001
Aging * Thickness	47.442	2	23.721	15.63	<0.001
Aging * Heat	294.23	2	147.115	96.934	<0.001
Aging * Surface	334.746	1	334.746	220.564	<0.001

### Analysis of upper surfaces

The hardness values of the upper surfaces and changes after aging are as shown in Tables 3 and 4**.** In the GCP group, the unvarnished samples applied with thermo-light curing for 90 s gave statistically higher results than the varnished samples (*p* < 0.05). The highest GCP microhardness value was recorded in the unvarnished (88.1 ± 7.59) group which was thermo-light cured for 90 s. The thermo-light curing treatment increased the microhardness values of the unvarnished samples both before and after aging (*p* < 0.05) (Table 3). In the non-exposed samples of the GCP group, there was a statistically significant difference between the varnished and unvarnished samples after aging (*p* < 0.05) (Table 3).

**Table 3:** Upper surfaces; distribution of measurements according to material, heat and varnish application state

Material	Heat	Varnish	Before Aging		After Aging	
GCP	Non-exposed	Varnished	77.67±6.35	a,x	64.91±6.31	a,y
		Unvarnished	72.42±10.61	a,x	58.93±10.97	b,y
	60 sec	Varnished	81.94±6.49	a,x	70.94±6.55	a,y
		Unvarnished	81.89±5.22	a,x	69.4±5.01	a,y
	90 sec	Varnished	79.47±4.25	a,x	71.34±4.28	a,y
		Unvarnished	88.1±7.59	b,x	77.33±7.91	b,y
Equia Forte	Non-exposed	Varnished	125.6±6.79	a,x	118.35±6.82	a,y
		Unvarnished	120.12±9.9	a,x	111.87±9.99	b,y
	60 sec	Varnished	110.28±13.26	a,x	105.95±12.01	a,y
		Unvarnished	120.96±9.05	b,x	114.13±9.06	b,y
	90 sec	Varnished	112.22±13.23	a,x	108.22±13.27	a,y
		Unvarnished	123.83±8.12	b,x	118.99±8.19	b,y

(a, b: column comparison / x, y: row comparison). (a, b and x, y): The same letters indicate an insignificant difference among the groups (p < 0.05). The a and b letters indicate the relationships between the two lines, where measurements based on varnish variations are specified. It does not specify the relationships among the groups in terms of temperature or material differences

**Table 4:** Upper surfaces; distribution of measurements according to material, varnish, and heat application state

Material	Varnish	Heat	Before Aging		After Aging	
GCP	Varnished	Non-exposed	77.67±6.35	a,x	64.91±6.31	a,y
		60 sec	81.94±6.49	a,x	70.94±6.55	a,y
		90 sec	79.47±4.25	a,x	71.34±4.28	a,y
	Unvarnished	Non-exposed	72.42±10.61	a,x	58.93±10.97	a,y
		60 sec	81.89±5.22	b,x	69.4±5.01	b,y
		90 sec	88.1±7.59	b,x	77.33±7.91	c,y
Equia Forte	Varnished	Non-exposed	125.6±6.79	a,x	118.35±6.82	a,y
		60 sec	110.28±13.26	b,x	105.95±12.01	b,y
		90 sec	112.22±13.23	b,x	108.22±13.27	b,y
	Unvarnished	Non-exposed	120.12±9.9	a,x	111.87±9.99	a,y
		60 sec	120.96±9.05	a,x	114.13±9.06	b,y
		90 sec	123.83±8.12	a,x	118.99±8.19	c,y

(a, b, c: column comparison / x, y: row comparison). (a, b and x, y): The same letters indicate an insignificant difference among the groups (p < 0.05). The a, b and c letters indicate the relationships between the three lines, where measurements based on heat variations are specified. It does not specify the relationships among the groups in terms of varnish or material differences

In the non-exposed samples of the EF group, there was a statistically significant difference between the varnished and unvarnished samples after aging (*p* < 0.05; Table 3). The highest EF microhardness value was recorded in the non-exposed-varnished group (125.6 ± 6.79). Although the thermo-light curing treatment increased the microhardness values of the unvarnished samples, it decreased the values of the varnished samples (*p* < 0.05). (Table 4). For EF material, the positive effect of thermo-light curing was better observed in the unvarnished samples.

### Analysis of bottom surfaces

The hardness values of the bottom surfaces of the samples are shown in Table 5. It is necessary to consider the difference in thicknesses in the evaluation of the bottom surfaces of the samples. In the non-exposed samples of the GCP group, there were statistically similar results at different thicknesses (*p* > 0.05). The bottom surface hardness values of the GCP group, which was thermo-light cured for 60 s and 90 s, statistically decreased as thickness increased (*p* < 0.05). After applying thermo-light curing to the samples in the GCP group, there were statistically significant differences between the 2- and 6-mm-thick groups (*p* < 0.05). They showed statistically similar results between 2 and 4 mm and 4–6 mm (*p* > 0.05; Table 5). The EF group was less affected by thermo**-**light curing than the GCP group. There were no significant differences in the hardness values of different thicknesses in the non-exposed groups which were thermo-light cured for 90 s (*p* > 0.05; Table 5).

**Table 5:** Bottom surfaces; distribution of measurements according to material, thickness and heat application state

Material	Heat	Thickness	Microhardness	
GCP	Non-exposed	2 mm	65.67±8.19	a
		4 mm	63.63±7.01	a
		6 mm	65.17±6.84	a
	60 sec	2 mm	67.71±7.71	a
		4 mm	62.28±9.46	ab
		6 mm	58.53±6.31	b
	90 sec	2 mm	70.61±8.83	a
		4 mm	62.8±9.05	ab
		6 mm	56.84±10	b
Equia Forte	Non-exposed	2 mm	112.35±11.53	a
		4 mm	110.92±8.72	a
		6 mm	112.04±7.99	a
	60 sec	2 mm	116.82±10.11	a
		4 mm	111.18±11.48	ab
		6 mm	104.04±17.83	b
	90 sec	2 mm	113.66±14.24	a
		4 mm	109.83±6.52	a
		6 mm	105.94±9.85	a

(a, b: column comparison). (a, b): The same letters indicate an insignificant difference among groups (p < 0.05). The a and b letters indicate the relationships between the three lines, where measurements based on thickness variations are specified. It does not specify the relationships among the groups in terms of heat or material differences

### SEM images of surface topography

The SEM images obtained from the upper surfaces of the GCP and EF samples according to varnish and thermo**-**light curing procedures are shown in Figs. 1, 2, 3, 4, 5, 6, 7, 8, 9, 10, 11 and 12, respectively.

**Fig. 1:**
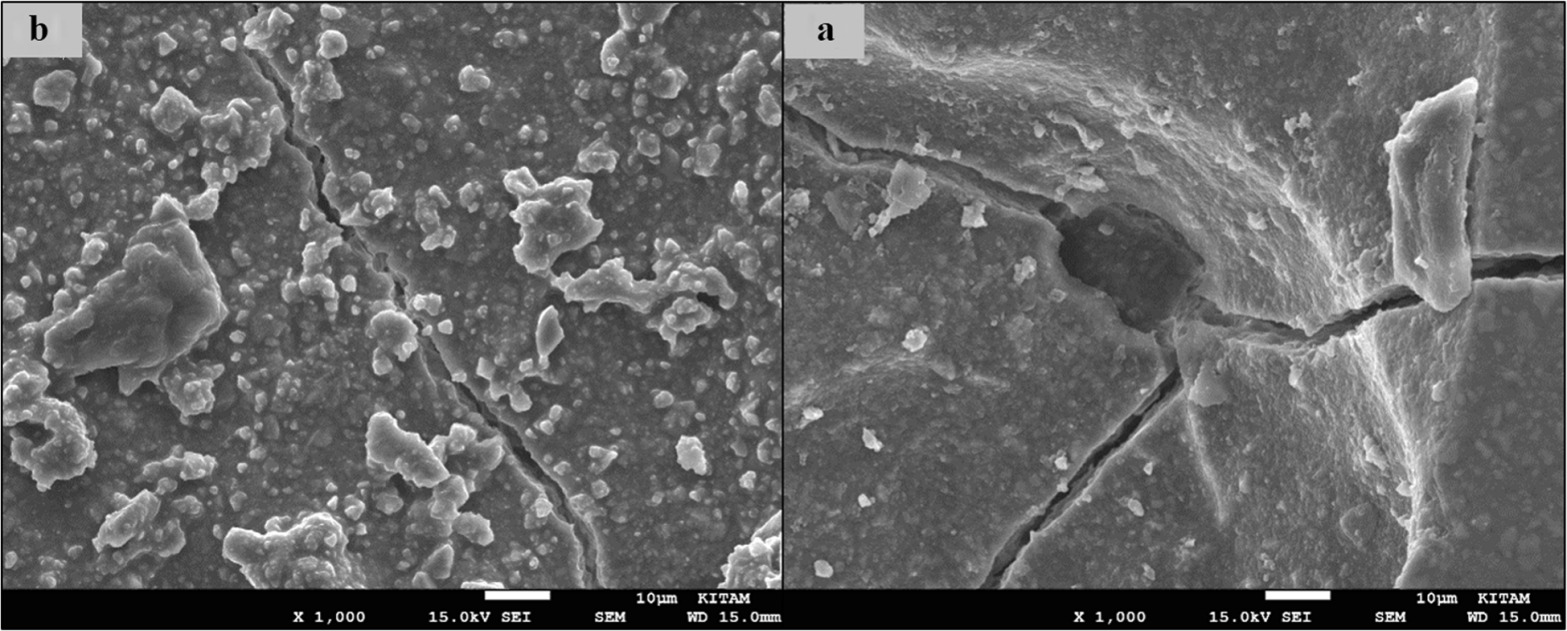
SEM images of varnished GCP samples before(**b**) and after(**a**) thermal aging in × 1000 magnification and heated by non-exposed

**Fig. 2:**
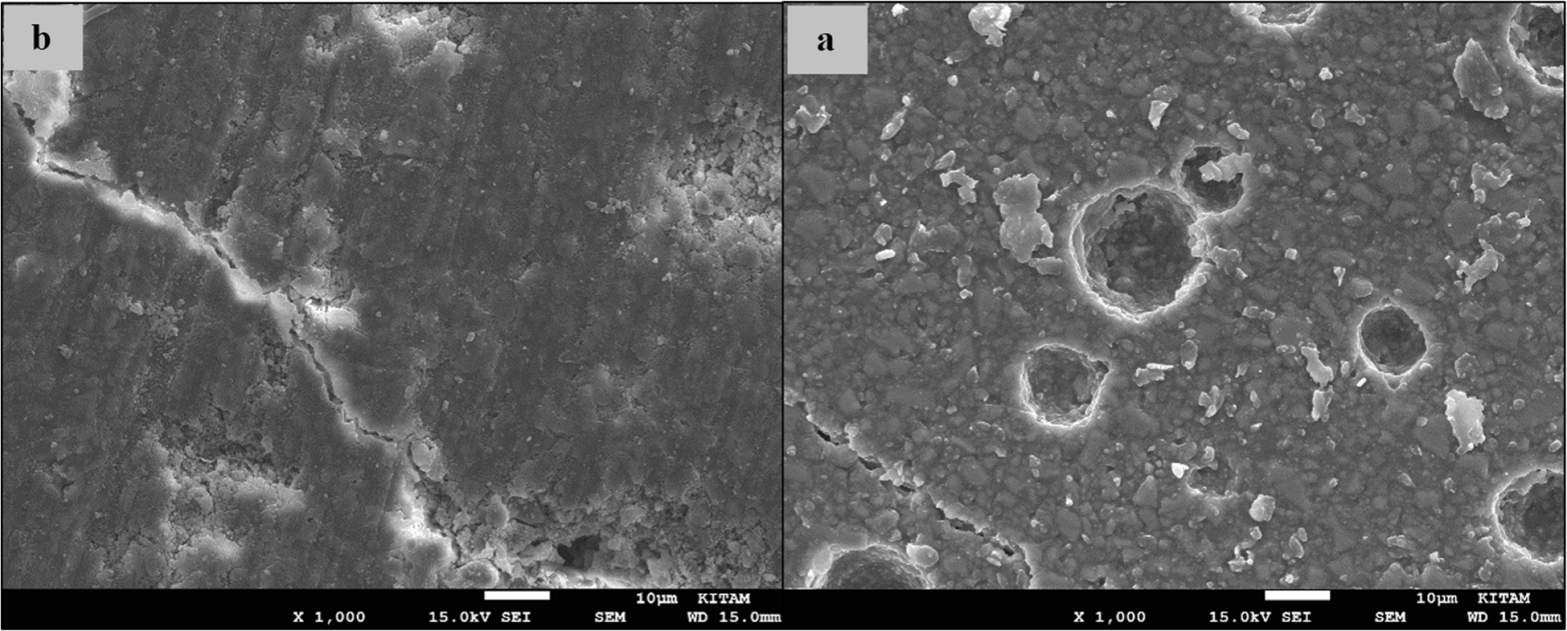
SEM images of varnished GCP samples before(**b**) and after(**a**) thermal aging in × 1000 magnification and heated by 60s

**Fig. 3:**
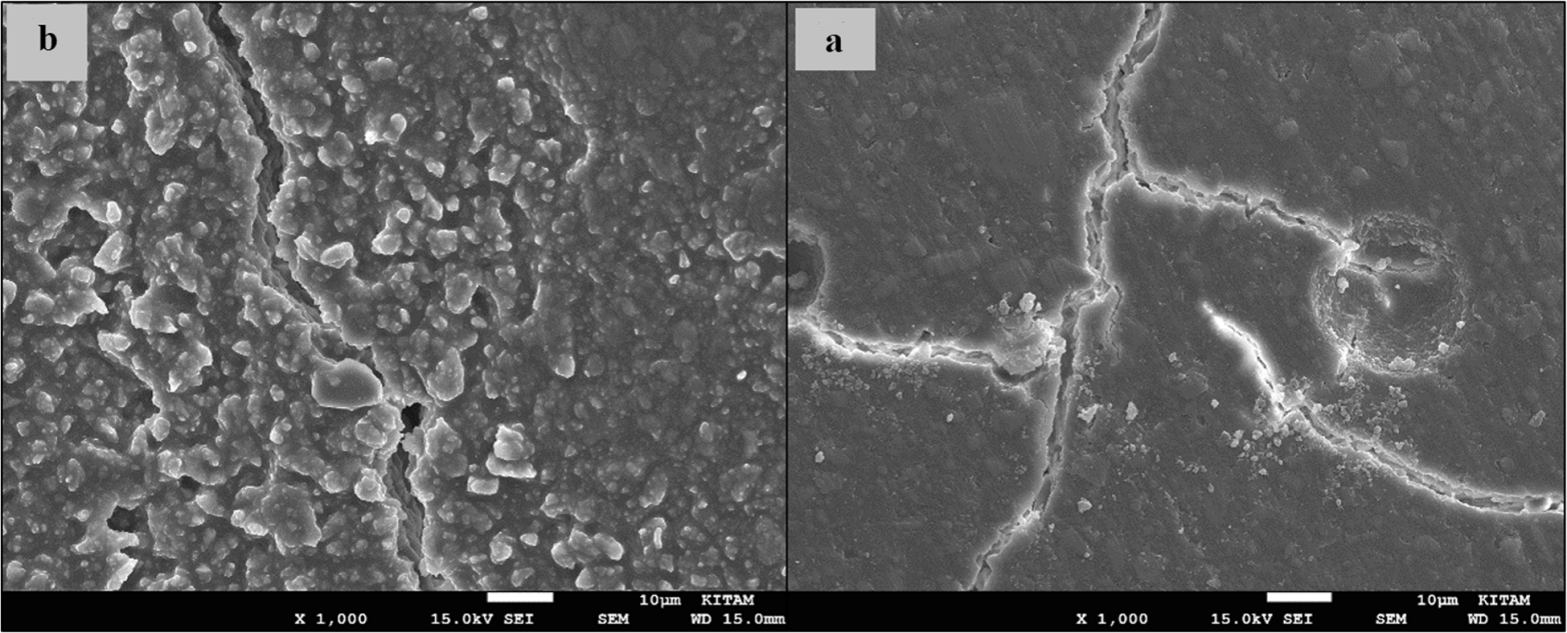
SEM images of varnished GCP samples before (**b**) and after (**a**) thermal aging in × 1000 magnification and heated 90s

**Fig. 4:**
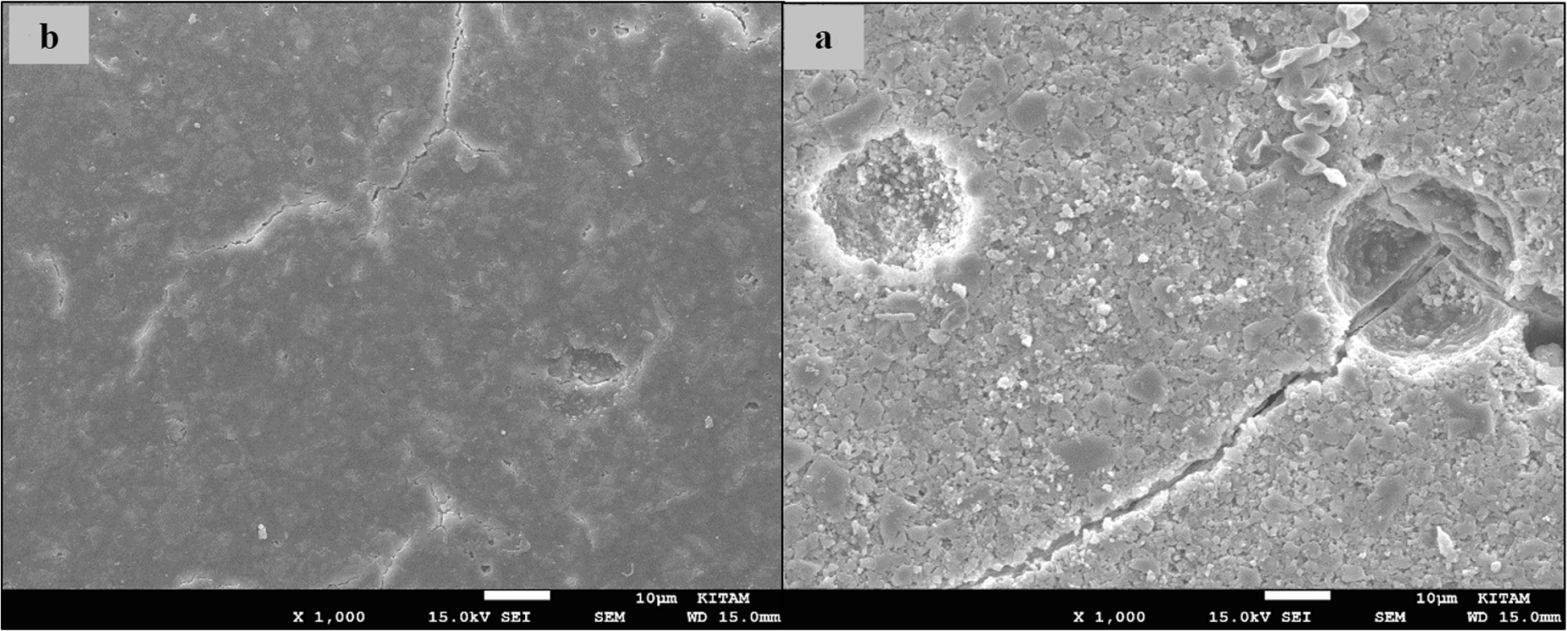
SEM images of non-varnished GCP samples before(**b**) and after(**a**) thermal aging in × 1000 magnification and heated by non-exposed

**Fig. 5:**
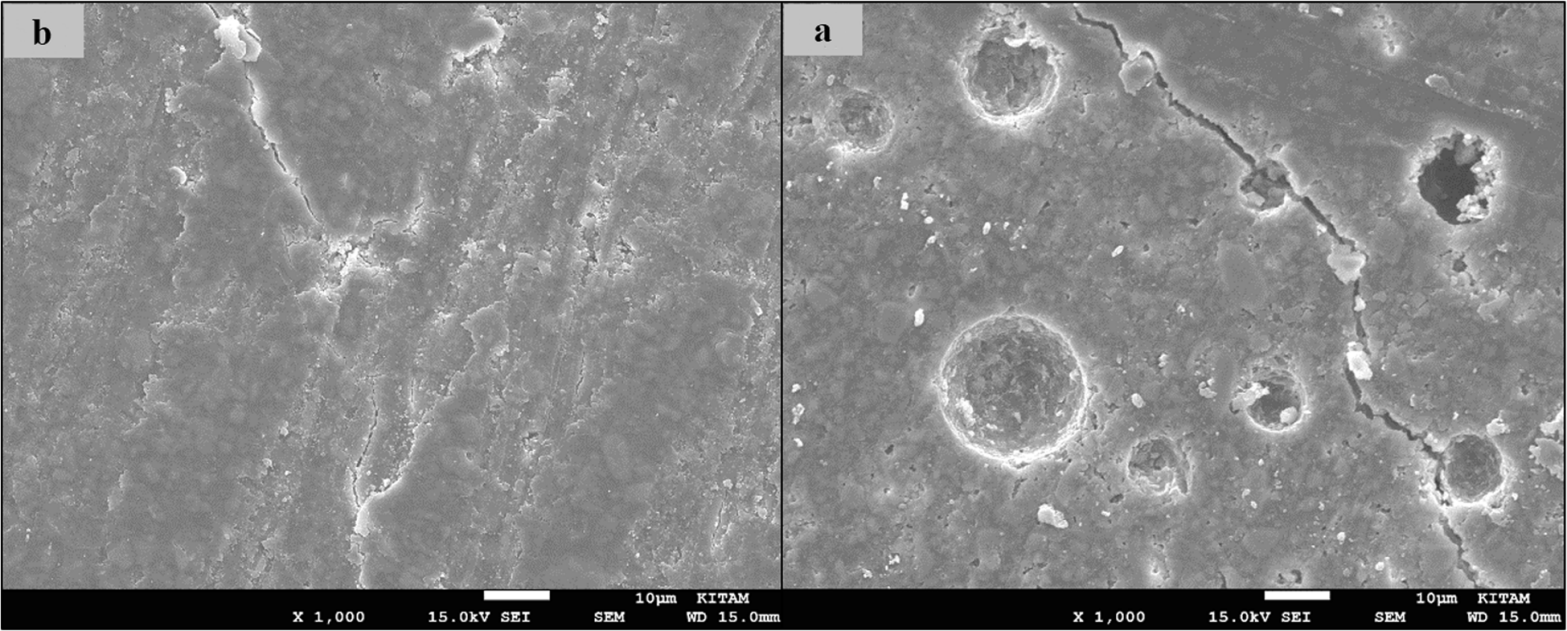
SEM images of non-varnished GCP samples before(**b**) and after(**a**) thermal aging in × 1000 magnification and heated by 60s

**Fig. 6:**
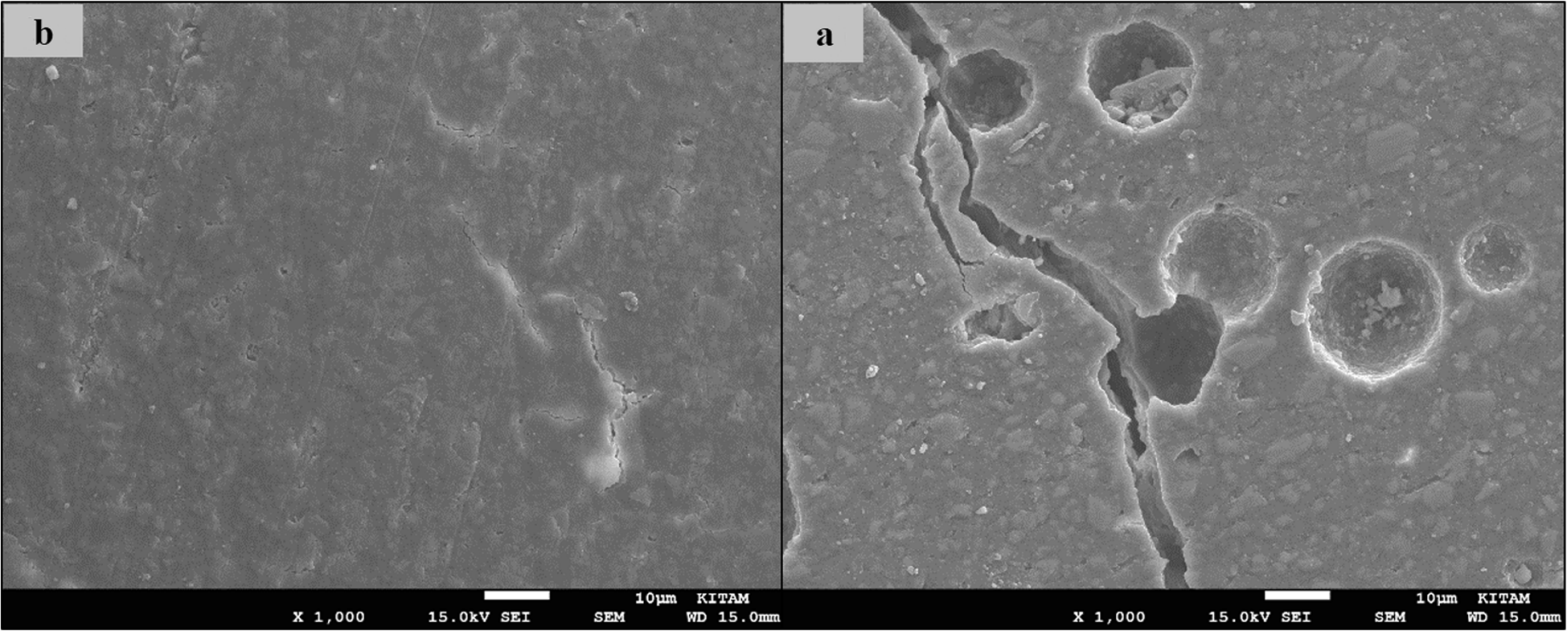
SEM images of non-varnished GCP samples before(**b**) and after(**a**) thermal aging in × 1000 magnification and heated by 90s

**Fig. 7:**
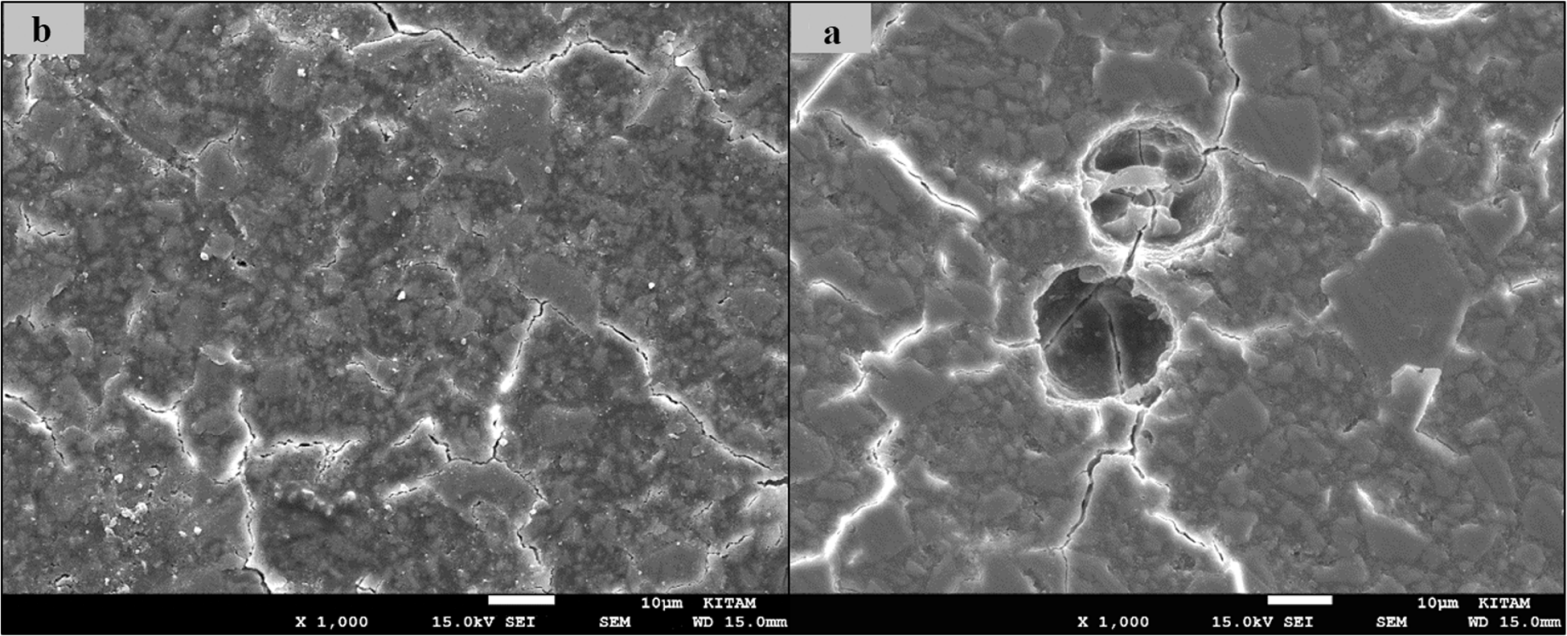
SEM images of varnished EF samples before(**b**) and after(**a**) thermal aging in × 1000 magnification and heated by non-exposed

**Fig. 8:**
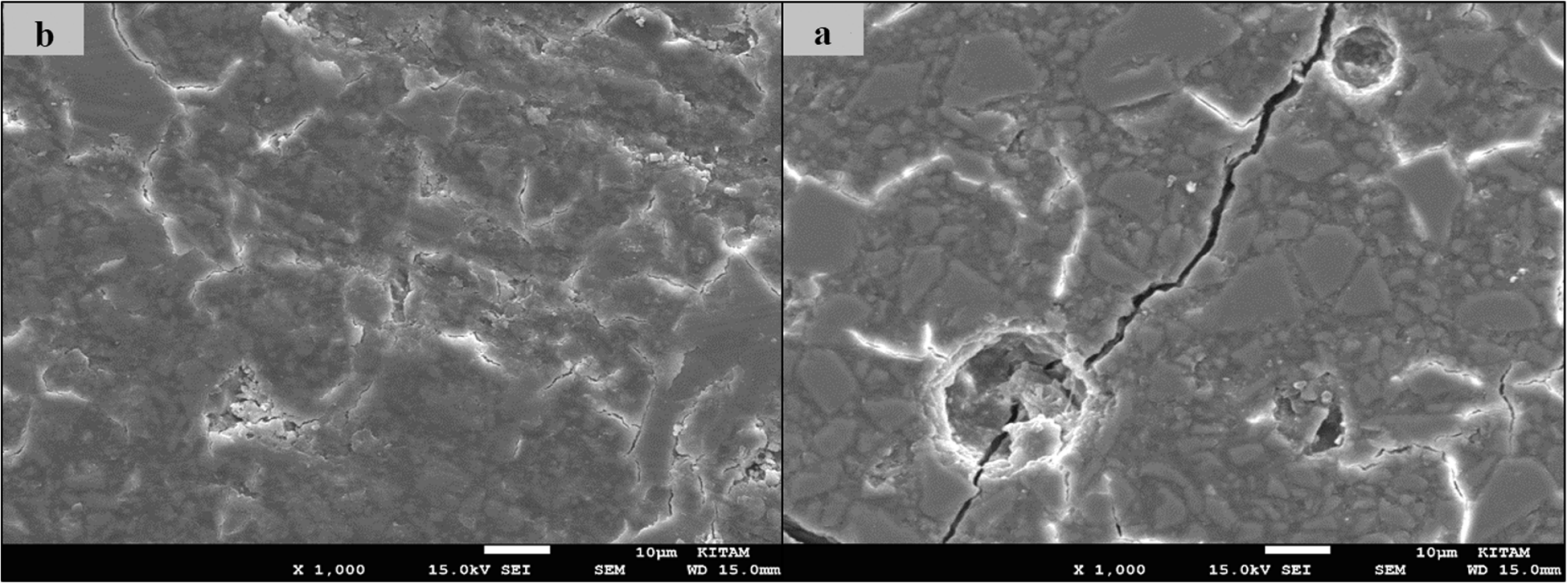
SEM images of varnished EF samples before(**b**) and after(**a**) thermal aging in × 1000 magnification and heated by 60s

**Fig. 9:**
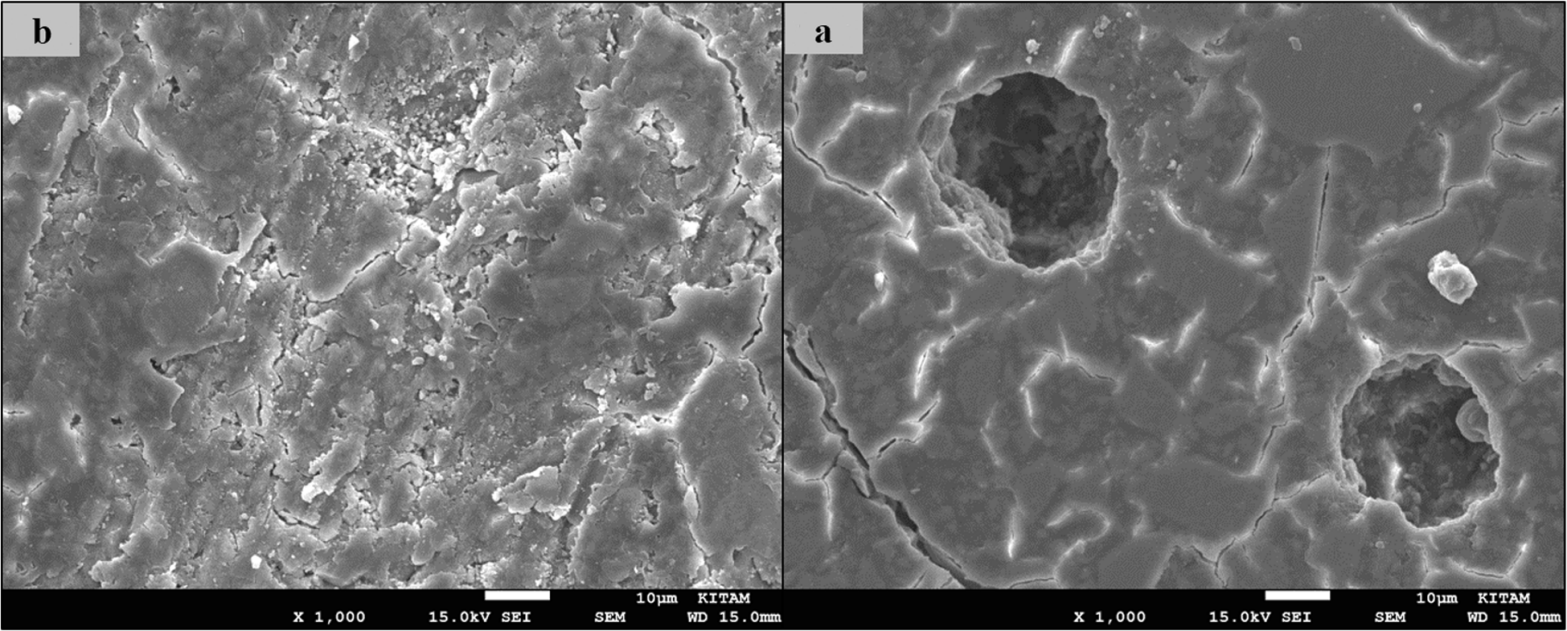
SEM images of varnished EF samples before(**b**) and after(**a**) thermal aging in × 1000 magnification and heated by 90s

**Fig. 10:**
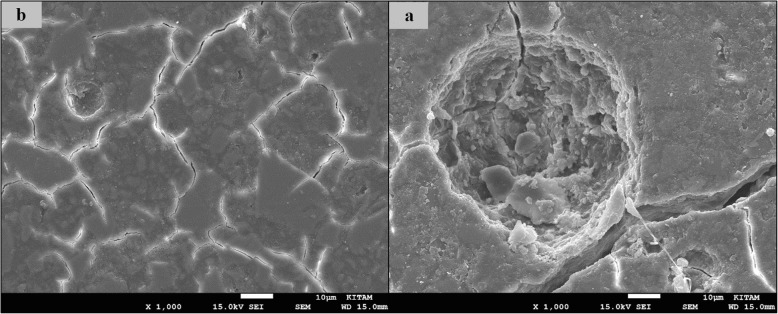
SEM images of non-varnished EF samples before(**b**) and after(**a**) thermal aging in × 1000 magnification and heated by non-exposed

**Fig. 11:**
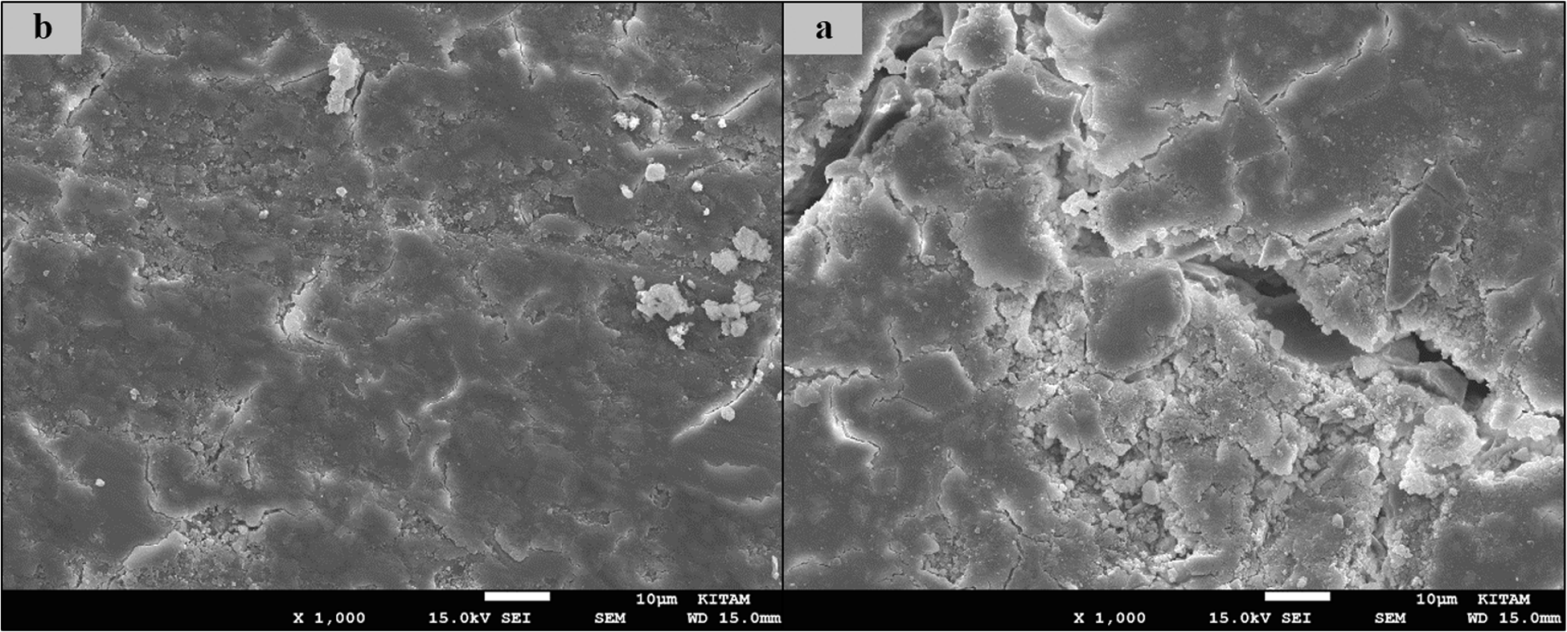
SEM images of non-varnished EF samples before(**b**) and after(**a**) thermal aging in × 1000 magnification and heated by 60s

**Fig. 12:**
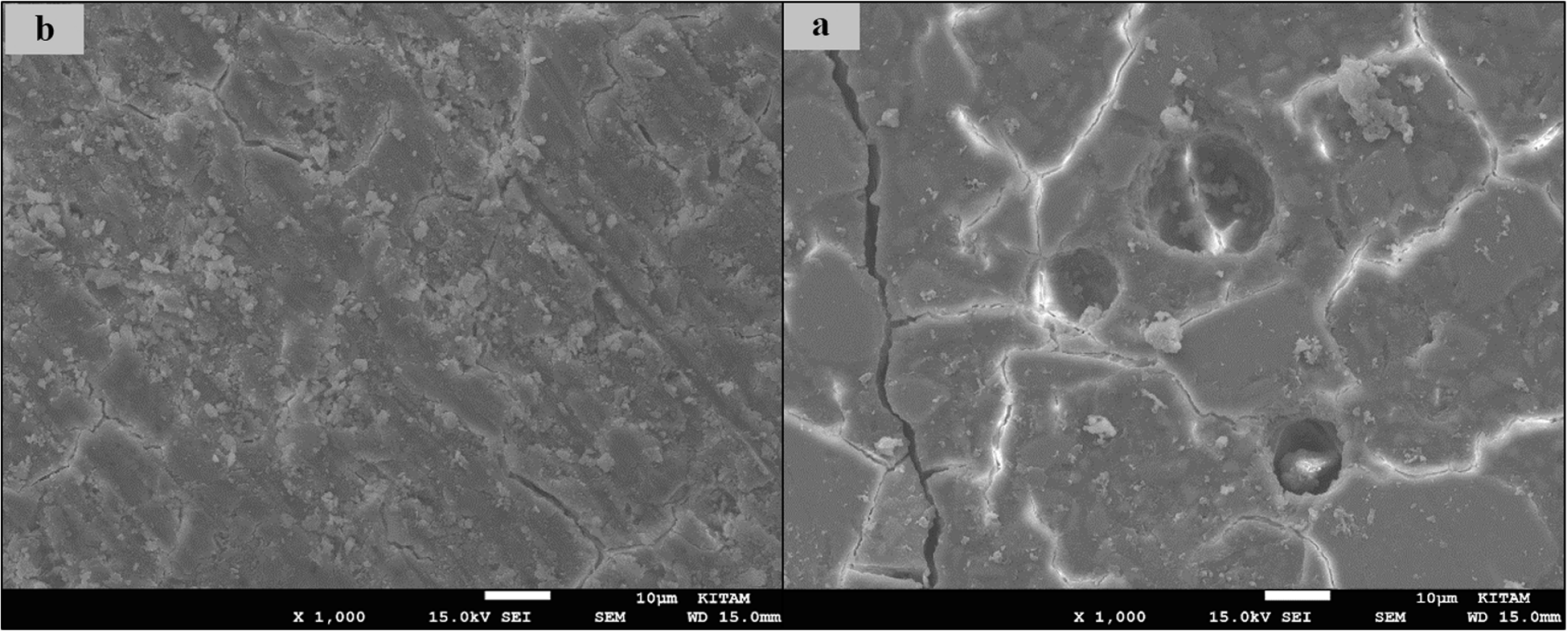
SEM images of non-varnished EF samples before(**b**) and after(**a**) thermal aging in × 1000 magnification and heated by 90s

When the SEM images of the GCP and EF samples (× 1000 magnifications) were evaluated, the unvarnished GCP and EF samples, which were thermo-light cured for 90 s, showed the smoothest SEM images according to the other samples before aging. These groups showed the highest hardness values within themselves when evaluated before aging. This result was especially observed in the GCP samples. The same groups (the unvarnished GCP and EF groups which were thermo-light cured for 90 s) had deep cracks, pits, and fissures after aging.

The varnished GCP and EF samples applied for 90 s with light gave more irregular crater and crack images than the unvarnished samples before aging. Small debris was observed on the surface of the varnished samples of nonaging GCP. Varnish application also caused surface roughness and cracks in the samples of the EF group. The surface roughness and cracks were greater in the non-exposed EF samples than in the EF samples which were thermo-light cured for 90 s. The sample deformations and cracks after aging were greater than before in both materials. Applying thermo-light curing in 90 s to the samples reduced the cracks in both the GCP and EF groups before and after aging (Figs. 1, 2, 3, 4, 5, 6, 7, 8, 9, 10, 11 and 12).

## Discussion

Brinell, Rockwell, Berkovich, Knoop and Vickers test methods are the four standards for measuring the hardness of the material. The microhardness of materials can be measured by various methods, and the Vickers micro-indentation test is one of the most commonly used tests. The trace produced by the Vickers identifier is clearer than that of other tests [26]. The microhardness test method is used when samples are small or thin. The 136-degree diamond pyramid-shaped indenter is applied to the material with the specified time and force. The hardness values of different filling materials measured in the study were statistically different from each other (Table 2), (*p* < 0.05). Therefore, we rejected our first null hypothesis, “There would be no difference between the two different restorative materials to be used in the study in terms of surface hardness values before and after thermal aging”, because the hardness values of the samples in the EF group before and after aging were statistically higher than those of the GCP group.

Conventional GICs can be thermo-cured by heat application using dental polymerization lamps, which causes acceleration in the setting. This acceleration leads to better mechanical properties, especially early mechanical properties, and decreases marginal leakage [27]. Kuter et al. reported that the upper surfaces of the GIC samples were more affected by heat and that the heat treatment increased the microhardness of GIC [28]. In their study, it was reported that heat application positively affected the mechanical properties of conventional GICs [9]. Heat application is supposed to accelerate the matrix-forming reaction of GCP [29]. Present findings for GCP have shown that thermo-light curing with power outputs of 1000 and 1200 mW/cm^2^ is required to achieve the desired high flexural strength of the material [20]. It was reported that raising the temperature of the surface of the cement to a maximum of 60 °C significantly improved the surface hardness of the material after 24 h [18]. Menne-Happ reported that heat treatment and gloss application did not influence the mechanical properties of GCP [30]. In our study, we observed that the surface hardness values of the unvarnished GCP samples increased. These findings are parallel to the previous studies [9, 31]. In the present study, the hardness values of the GCP and EF samples were examined before and after thermocycling aging. The varnished EF samples showed statistically different hardness values in different thermo-light curing procedures. Only in varnished EF samples, thermo-light curing had an adverse effect on surface hardness, whereas in other groups (GCP and EF) thermo-light curing increased the surface hardness. According to these results, our second null hypothesis, “Different thermo-light curing procedures would not change the surface hardness values of the GCP and EF samples”, was rejected. The different response to thermo-light curing application may be due to the different contents of the two materials.

Surfacing with resin-based varnishes not only protects initial water contamination but also increases the physical properties and wear resistance of the material. On the one hand, using GCP Gloss protects the material from desiccation. On the other hand, it helps model and polish fillings. It is also biocompatible and 100% monomer-free (http://gcp-dental.com/products/gcp-gloss/). According to the manufacturer, GCP Gloss is a silicone-based coat to protect the surface from exposure to moisture and saliva during the first setting reaction and dehydration in the second phase. In many studies, resin-based, light-active surface-coating agents have been found to perform better than other types of surface-coating agents [14, 32]. Again an in vitro study reported that the application of GCP Gloss did not improve the mechanical properties of GCP [30]. In their in vivo study, Gurgan et al. reported that Equia Fil had an additional advantage when compared to GCP and Equia Forte Coat, which, with a light-cured, nanofilled, resin-based coat, infiltrated the surface and margins of the restoration, improved the resistance to abrasion and reduced the marginal microfracture of the restoration [33]. A clinical trial on permanent teeth reported that Equia Fil with a resin-based coat showed better performance in class I and II restorations when compared to Fuji IX GP with LC Coat [34]. Another in vivo study reported that the longevity of glass carbomer atraumatic restorative treatment class II restorations was inferior to that of high-viscosity GI restorations after 12 months of clinical service in primary teeth [35].

In the EF group of the present study, the varnish in the non-exposed samples did not make a significant difference in the surface hardness values. However, after thermal aging, the non-exposed varnished EF samples showed higher surface hardness values in comparison to the unvarnished samples. This result indicates that varnish application positively affects the moisture sensitivities of the EF samples. In the samples hardened by thermo-light curing for 60 and 90 s, the varnished samples gave a lower surface hardness as compared to the unvarnished ones before and after aging. The reason for this may be that the Equia Forte Coat varnish is resin-based and contains camphorquinone. The light device we used may not have been able to polymerize without having the light output at the wavelength required for varnish polymerization. Accordingly, the methyl methacrylate in the nonpolymerized varnish may have reduced the surface hardness of groups subjected to thermo-light cured for 60 and 90 s with a solvent effect. Different results were obtained in the varnished GCP samples prepared to prevent the moisture sensitivity of GIC. If GCP was self-hardened without thermo-light curing, the samples with and without varnish showed statistically similar results. However, after thermal aging, the non-exposed and varnished GCP samples showed higher surface hardness results than the unvarnished samples. This result indicates that varnish application positively affects the moisture sensitivities of GCP. In the samples hardened by thermo-light cured for 90 s, the varnished samples gave a lower surface hardness value than that of the unvarnished ones before and after aging. This result of our study was parallel to the study conducted by Menne-Happ. Menne-Happ reported that heat treatment and gloss application did not influence the mechanical properties of GCP. While the GCP polymer does not contain monomers and consists of modified polysiloxanes, varnishes used to protect the conventional GIC are mostly acrylic or methacrylic monomers that can be polymerized according to the manufacturer [30].

In a recent study, in the control groups of Equia Fil, there were no statistically significant differences about surface roughness between the samples treated with thermo-curing and the samples without thermo-curing [36]. In the current study, the SEM images obtained from the upper surfaces of the EF samples are shown in Figs. 7, 8, 9, 10, 11 and 12. In the EF group that was varnished and thermo-light cured for 90 s, surface cracks and erosive areas were observed before and after thermal aging. Surface morphologies gave similar appearances before thermal aging. However, there are morphological differences after aging. Cracks and craters were observed in all the EF samples especially after aging.

In the present study, the SEM images of GCP are shown in Figs. 1, 2, 3, 4, 5 and 6. The surfaces of the varnished GCP samples had a particulate structure before aging, which disappeared after aging. In the samples, which were varnished and thermo-light cured for 90 s, surface cracks were observed both before and after thermal aging. When the GCP samples were not varnished, the surface morphology was smoother, and there was no structure of any particulate. There were erosive areas in all aged GCP samples. It was reported in a study that the number of voids and cracks was generally greater for GCP samples than for resin-modified glass ionomer cement (RMGIC) and that heat treatment and gloss application did not influence the mechanical properties of GCP [30]. The SEM images of the present study showed a result parallel to the study conducted by Menne-Happ.

The bottom surface hardnesses of the 2, 4, and 6-mm-thick GCP and EF samples were evaluated in the present study. As the sample thicknesses increased for GCP and Equia Forte materials, the bottom surface hardness decreased. The highest bottom surface hardness values of the GCP samples was observed in the 90 s thermo-light-cured sample of 2 mm thickness (70.61 ± 8.83). It is considered that the non-exposed sample of 6 mm thickness will give the lowest surface hardness result. However, the lowest hardness value was observed in the sample of 6 mm thickness which was thermo-light cured for 90 s (56.84 ± 10.00). This indicates that the thermal conductivity of GCP is different from that of the conventional GIC.

According to the manufacturer’s instructions, GCP fıll is a new carbomised, nanoparticle-containing GI restorative cement with a specially designed filler and fluorapatite/hydroxyapatite particles for reduced solubility. In other words, GCP Gloss fıll does not contain any resins, solvents, and metals and is monomer-free (http://gcp-dental.com/products/gcp-gloss/). In their in vitro study, Menne-Happ and Ilie reported that, in the SEM analysis, the RMGICs, Photac Fil (3 M ESPE AG, Germany), and Fuji II LC showed a similar surface morphology: large glass particles were observed in the microstructures compared to GCP [30].

When we evaluated the GCP samples according to their thicknesses, the thermo-light curing change affected the 6-mm-thick samples more than others. While there were no differences among the hardness values of different sample thicknesses in the non-exposed GCP samples, the hardness values decreased in the samples which were thermo-light cured for 60 and 90 s as the thickness increased. (Table 5).

In a similar study, Equia Fil samples with different thicknesses (2,3,4 mm) were prepared. The control group, which is non-exposed, and three groups, which are thermo light-cured with three different light devices, were formed. The differences between the microhardness values of the samples were statistically significant [12].

The properties of thermal conductivity are important if sufficient heat is transferred from the surface of the cement, where the thermo cure light is applied to the bottom [19]. In their in vitro study, Gavic et al. evaluated the heat transfer properties of GICs at different thicknesses (2, 3, and 4 mm). They reported that the glass component had a higher thermal conductivity than the matrix and that the thermal conductivities of the three different cements were similar [19]. In both EF and GCP materials of the current study, the effect of temperature variables on the bottom surface hardness of each thickness group was not significant. In the non-exposed samples, the thickness variations of both materials did not change the bottom surface hardnesses (Table 5). In the samples that were thermo-light cured, the bottom surface hardness values decreased in all the groups as the thicknesses increased in both materials (EF and GCP). The thermo-light curing increase affected the 2-mm-thick samples more than others. The positive effect of thermo-light curing was not observed in the samples thicker than 2 mm (Table 5). We think that this is related to the contents and thermal conductivities of the materials. As with all in vitro studies, the findings of the present study should be tested in clinical settings in future clinical trials. The clinical success of the materials should be evaluated in the future with randomized controlled studies.

## Conclusion

Within the limitations of this research, the following conclusions were drawn:

- Thermal aging adversely affected the microhardness of the materials, which is important for clinical success.
- Thermo light curing process improved the microhardness of the GCP group without varnish application. For this material, the highest value was observed in the thermo-light-curing process for 90 s without varnish. This application may be recommended to the clinical dentistry.
- Varnish application increased the microhardness of the EF group without applying thermo-light curing. Therefore, the combination of external thermal curing and varnish may not be suggested for EF cement in clinical practices.
- The microhardness of the bottom surfaces decreased with increasing thickness. The thermo-light curing did not increase the bottom surface microhardness of the samples for EF and GCP.

## Data Availability

The datasets used and/or analyzed during the current study are available from the corresponding author on reasonable request.

## Abbreviations

Å: Ångström
EF: Equia Forte
GCP: Glass Carbomer Cement
GI: Glass Ionomer
GIC: Glass Ionomer Cement
HV: Hardness Value
mN: microNewton
RMGIC: Resin Modified Glass Ionomer Cement
SEM: Scanning Electron Microscope
VHN: Vickers Hardness Number
